# Efficacy of topical application of simvastatin gel combined with a collagen sponge carrier in preserving alveolar ridge dimensions: a triple-blind randomized clinical trial

**DOI:** 10.1007/s00784-026-06832-9

**Published:** 2026-03-25

**Authors:** Elena López-Andrade, Francisco Javier Manzano-Moreno, Virginia Taboada, Cristina Vallecillo, Laura Jiménez-Andújar, Marta Vallecillo-Rivas

**Affiliations:** 1 Faculty of Dentistry, Colegio Máximo de Cartuja s/n, University of Granada, Granada, 18071 Spain; 2 Medicina Clínica y Salud Pública PhD Programme, Faculty of Dentistry, University of Granada, Granada, 18071 Spain; 3 Department of Stomatology, Faculty of Dentistry, University of Granada, Granada, 18071 Spain

**Keywords:** Simvastatin, Alveolar ridge preservation, Randomized clinical trial, CBCT, Vertical bone loss, Third molar, Bone density

## Abstract

**Background:**

Alveolar ridge atrophy is an inevitable consequence following tooth extraction, leading to substantial vertical and horizontal bone loss and frequently complicating subsequent prosthetic rehabilitation. Simvastatin (SIM), known for its osteoinductive and anti-inflammatory properties, has shown promise in promoting local bone regeneration. This study aimed to evaluate the efficacy of topical application of a novel 1.2% SIM gel in preserving alveolar ridge dimensions following the surgical extraction of mandibular third molars, compared with a placebo.

**Methods:**

A randomized, triple-blind clinical trial (RCT) was conducted involving 40 patients (*n* = 20 per group) requiring mandibular third molar extraction. Patients were randomly assigned to receive either the active 1.2% SIM gel (test group) or a placebo gel (control group). In both groups, the assigned gel was delivered using a collagen sponge as a carrier scaffold placed directly into the extraction socket. Cone Beam Computed Tomography (CBCT) scans were obtained pre-extraction (T0) and 12 weeks postoperatively (T1). The primary outcome was the dimensional change in vertical alveolar bone height (ΔH). Secondary and exploratory outcomes included changes in bone width (ΔW), bone density (Mean Gray Value), postoperative pain, and swelling.

**Results:**

The primary outcome analysis included 38 sockets (*n* = 19 per group). The Test Group (SIM) showed significantly greater vertical bone preservation, with a mean change in height of − 0.30 ± 1.79 mm (indicating preservation/gain), compared to the Control Group’s mean loss of 1.28 ± 1.50 mm (*p* = 0.006). No statistically significant difference was observed in exploratory bone width preservation (*p* > 0.05). The SIM gel demonstrated an excellent safety profile, with no significant differences regarding postoperative pain or swelling compared to the placebo.

**Clinical Significance:**

The topical application of 1.2% SIM gel significantly improved vertical alveolar bone height preservation at 12 weeks compared to the placebo. This primary finding confirms the clinical benefit of local simvastatin delivery in maintaining the vertical dimension required for successful prosthetic treatment, while exploratory densitometric data also suggested an improvement in coronal bone mineralization. This simple, low-cost pharmacological approach is highly effective in mitigating post-extraction vertical bone loss.

## Introduction

Tooth extraction initiates a cascade of cellular responses that result in significant three-dimensional remodeling of the alveolar bone [1]. This inevitable post-extraction resorption leads to a substantial loss of bone height and width [2, 3]. Clinically, major studies report that vertical and horizontal bone loss can range from 11 − 22% and 29 − 63%, respectively, within the first six months [4, 5]. Crucially, the reported loss of approximately 50% of the alveolar ridge width severely compromises future dental treatments, particularly the successful placement of dental implants and subsequent prosthetic rehabilitation [4–6].

To counteract this, Alveolar Ridge Preservation (ARP) therapies have been widely adopted to attenuate post-extraction resorption [4, 7]. The standard ARP approach involves socket filling with various biomaterials, such as bone graft particles, which have proven effective compared to unassisted healing [4, 6]. However, the clinical applicability of conventional grafting techniques is often limited by factors such as complexity, high cost, and potential morbidity associated with these materials [8]. Seeking to reduce patient discomfort, risk of complications (up to 38% in some complex procedures [6, 9]) and lengthy treatment times [10], necessitating the search for simplified, less invasive, low-cost, and more predictable alternatives [8, 11]. In this context, pharmacological agents capable of accelerating tissue maturation and promoting endogenous bone regeneration represent a promising, minimally invasive clinical strategy.

Bone regeneration is regulated by growth factors, with Bone Morphogenetic Proteins (BMPs) playing a central role by promoting the osteogenic differentiation of mesenchymal stem cells [12, 13]. Therefore, pharmacological stimulation of BMP-2 expression is a valuable approach for enhancing local bone formation [14]. Simvastatin (SIM), primarily known as a cholesterol-lowering drug, has attracted attention in dentistry due to its pleiotropic effects, notably its powerful osteoinductive and antiresorptive properties [15, 16]. SIM inhibits 3 Hydrixy-3-methylglutaryl coenzyme A (HMG-CoA) reductase, a mechanism that has been consistently associated with increased expression of BMP-2 and Vascular Endothelial Growth Factor (VEGF) [17–20]. Furthermore, SIM exhibits anti-inflammatory effects and inhibition of osteoclast differentiation, making it an ideal local adjunct for post-extraction healing [19, 20].

Given its potent local effects, SIM has been increasingly utilized in oral and maxillofacial surgery, including for socket preservation and treatment of bone defects [21–24]. Crucially, due to its hepatoselective metabolism and limited systemic bioavailability, local drug delivery systems are clinically preferred to achieve high therapeutic concentrations directly at the bone regeneration site while mitigating the risk of systemic side effects [13, 23]. In recent years, SIM has been successfully incorporated into various biocompatible vehicles (such as gels and sponges) to ensure adequate drug retention and sustained, localized release [11, 24]. The topical application of simvastatin within these systems has shown promising results in various intraoral regenerative scenarios, including periodontal defects and sinus lift procedures [21, 25, 26].

Despite the compelling preclinical evidence and encouraging pilot studies [11, 23, 27–29], there remains a need for high-quality evidence, specifically triple-blind Randomized Clinical Trials (RCTs), to confirm the efficacy of SIM-based local delivery systems in preserving alveolar ridge dimensions.

Therefore, the aim of this study was to evaluate the efficacy of a novel 1.2% simvastatin gel, delivered via a collagen sponge carrier, in preserving vertical and horizontal alveolar ridge dimensions following mandibular third molar extraction, compared with a placebo gel delivered on an identical collagen sponge, in a triple-blind randomized controlled trial.

## Materials and methods

### Study design, registration, and ethics

This investigation utilized a triple-blind RCT design (two-group, parallel-group). The primary objective was to evaluate the efficacy of the 1.2% Simvastatin (SIM) gel in preserving alveolar bone after mandibular third molar extraction, specifically by comparing dimensional changes (bone Height and Width) against an identical placebo gel.

The RCT strictly followed the recommendations of the CONSORT 2010 Statement for reporting parallel group randomized trials [30] and its extension for nonpharmacologic treatments [31], taking into account the most recent updates on the guidelines CONSORT 2025 [32]. Furthermore, the risk of bias was controlled by methodological strategies considering “The Cochrane Collaboration’s tool for assessing the risk of bias in randomized trials. The study protocol, including the off-label local application of simvastatin, was reviewed and approved by the Institutional Ethics Committee of the University of Granada (Spain) (approval number: 3904/CEIH/2023), and all procedures were conducted under appropriate safety monitoring in accordance with ethical and regulatory standards, and registered in a public clinical trials registry (ClinicalTrials.gov Identifier: NCT07031778).

### Sample size and statistical power

The sample size calculation was performed exclusively based on the primary outcome variable, vertical bone height change (ΔH). Other parameters, including horizontal bone width change (ΔW), densitometric values, and clinical postoperative outcomes (pain, swelling, and trismus), were evaluated on an exploratory basis. Consequently, the study was not specifically powered to detect statistically significant differences for these secondary and exploratory parameters.

Based on previous randomized clinical trials evaluating the local application of simvastatin in extraction sockets [23, 24, 28]. Assuming a two-tailed α error of 0.05 and a statistical power (1 − β) of 80%, a minimum requirement of 18 patients per group was necessary to detect a clinically relevant difference. To compensate for potential loss to follow-up, the sample size was increased by 10%, leading to a final target of 20 patients per group, for a total sample size of *N* = 40 patients in the trial.

### Participants and eligibility criteria

The study employed a parallel group randomized clinical trial design. Inclusion and exclusion criteria were established to define the study population:

*Inclusion Criteria*:

1. Patients aged 18–45 years.
2. ASA I–II status according to the American Society of Anesthesiologists classification.
3. Indication for surgical extraction of at least one impacted mandibular third molar.
4. Absence of known allergy to study medications.
5. Provision of written informed consent.

*Exclusion Criteria* (Patients meeting at least one criterion were excluded):

1. Age outside the 18–45 range.
2. Chronic smoking (> 10 cigarettes/day).
3. ASA III–V status.
4. Current pharmacological treatment for hypercholesterolemia (systemic statin use).
5. Need for ostectomy extending beyond the Cementoenamel Junction (CEJ).
6. Noncompliance with study protocols or absence of written informed consent.

### Allocation, randomization, and blinding

Patients undergoing surgical extraction of impacted mandibular third molars were randomized by one of the researchers (M.V.-R.) who was not involved in the follow-up measurements. Random sequence generation was performed using a computer-generated random sequence (available at https://www.random.org/**).** Allocation was concealed using sealed and numbered opaque envelopes.

A total of 40 mandibular third molar sockets were randomly assigned into two study groups:

- Test group: Each socket received a 1.2% SIM gel combined with a collagen sponge carrier.
- Control group: Each socket was treated with a placebo gel (identical vehicle without SIM) combined with the same collagen sponge carrier.

The study maintained a triple-blind design, ensuring that the following parties were unaware of the group assignment: the patient, the operator performing the surgery and measurements (L.J.-A.), and the data analyst. The placebo and simvastatin gels were identical in color, texture, and smell, and were provided in identical syringes to ensure the blinding of the surgical operator.

### Simvastatin and placebo gel preparation and coding

The preparation of the gel and coding to ensure triple blinding were performed under the following protocol:

#### Polymeric vehicle (hydrogel) preparation

The base vehicle (hydrogel) was compounded by a local compounding pharmacy to ensure stringent quality control and sterility. The preparation of the 1.2% SIM gel followed the methodology described in previous clinical studies by Pradeep et al. [20–22, 33]. The formulation process involved mixing methylcellulose with a biocompatible solvent at 50–60∘C under continuous mechanical agitation until a homogenous polymeric vehicle was obtained.

#### Simvastatin gel (SIM 1.2%) preparation - test group

SIM powder (Sigma-Aldrich, St. Louis, MO, USA) was subsequently incorporated into the polymeric vehicle, achieving a final concentration of 12 mg/mL (1.2% formulation).

#### Placebo gel preparation - control group

The Placebo gel was produced using the identical compounding procedure as the Test group, but with the omission of Simvastatin.

#### Coding and blinding

Both formulations (SIM and Placebo) were supplied in coded, identical syringes. The gels were visually indistinguishable, preserving the triple-blinding of the study. The preparation was performed under aseptic conditions immediately prior to the surgical procedure.

### Surgical protocol and intervention

**Fig. 1:**
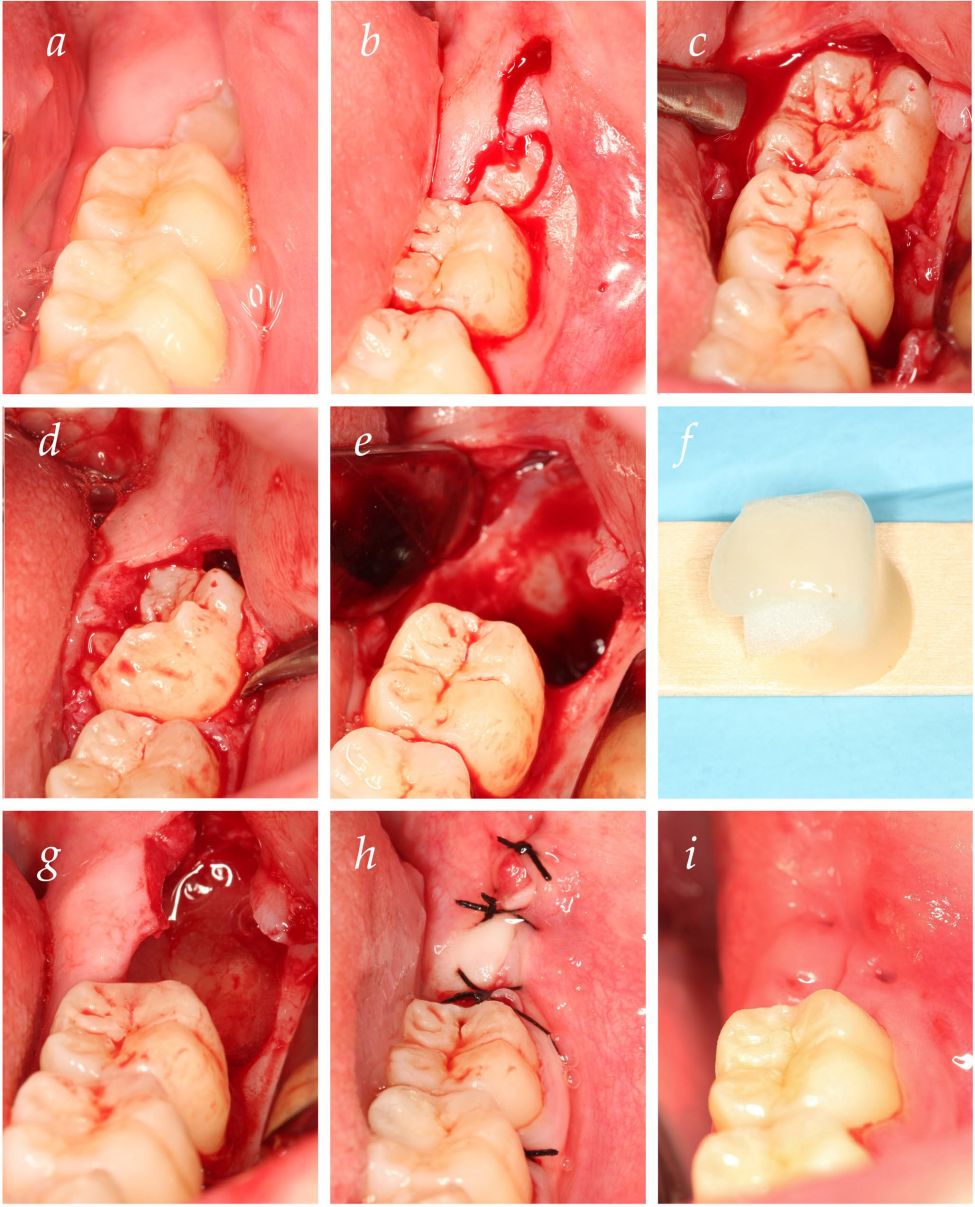
Surgical protocol. (**a**) Pre-surgical view; (**b**) bayonet incision; (**c**) elevation of a full-thickness flap and ostectomy up to the CEJ; (**d**) tooth luxation; (**e**) curettage and cleaning of the socket; (**f**) Preparation of the material (collagen sponge combined with gel); (**g**) placement into the extraction socket; (**h**) primary wound closure with sutures; (**i**) healing after suture removal at 7 days postoperatively

All surgical extractions of the impacted mandibular third molars were performed by a single, experienced oral surgeon (E.L.-A.G.) under standardized and sterile operating conditions to minimize procedural variability. The principal steps of the surgical technique and intervention are illustrated in Fig. 1.

Patients followed a standardized pre-operative protocol, including a one-minute rinse with 0.12% chlorhexidine mouthwash. Local anesthesia was achieved using 4% articaine with 1:100,000 epinephrine administered via conventional inferior alveolar nerve block and buccal infiltration. Anesthesia adequacy was confirmed prior to the surgical incision. The surgery was performed following a standardized technique in all cases. To access the third molar, either a linear or bayonet-shaped incision was made with a number 15 scalpel blade (Fig. 1b), followed by elevation of a full-thickness flap. Ostectomy was performed up to the CEJ using a straight handpiece and a number 3 bur (Fig. 1c). When necessary, odontosection was carried out using a high-speed turbine. After tooth luxation and tooth extraction (Fig. 1d), the site underwent meticulous curettage and irrigation with sterile saline solution to ensure thorough removal of all remnants of follicular tissue, debris, and granulation tissue, resulting in a clean, contained bony defect (Fig. 1e). After achieving hemostasis in the socket, the sealed, numbered envelope corresponding to the patient’s group assignment was opened by an assistant not involved in the surgical or follow-up procedures. The intervention material, consisting of a collagen sponge acting as a scaffold, was immediately combined with the coded gel (Fig. 1f) and applied as follows (Fig. 1g): in the test group the socket was filled with the 1.2% SIM gel (approx. 0.2 mL) using a blunt-tipped syringe until the gel reached the alveolar crest. In the control group the socket was filled with an identical volume of the placebo gel using the same technique. The mucoperiosteal flap was carefully repositioned and secured with interrupted sutures (3 − 0 silk or 4 − 0 resorbable material) to ensure primary wound closure (Fig. 1h) and effective retention of the study agent and scaffold. Sutures were removed at the 7-day follow-up visit (Fig. 1i).

All patients received a standardized post-operative medication regimen regardless of their assigned group. Analgesic management consisted of ibuprofen 400 mg administered orally every 8 h for 3 days. Prophylactic antibiotic therapy included amoxicillin 750 mg taken orally every 8 h for 7 days; in cases of documented penicillin allergy, clindamycin 300 mg was prescribed as an alternative. Additionally, patients were instructed to begin gentle rinsing with 0.12% chlorhexidine 24 h after the procedure, continuing twice daily for 7 days to support postoperative oral hygiene.

### Radiographic assessment and follow-up standardization

#### CBCT acquisition and technical parameters

CBCT scans were performed for each patient at baseline (T0) (immediately post-extraction and pre-gel application) and at 12 weeks post-extraction (T1) to assess changes in bone H and W [23, 29]. To ensure standardized image quality for both dimensional and densitometric analysis, all scans were performed using a Planmeca ProMax 3D unit (Planmeca Oy, Helsinki, Finland). The acquisition parameters were set to a field of view (FOV) of 100 × 100 mm and a voxel size of 200 μm (Normal resolution mode). Exposure settings were standardized at 120 kV and 6.3 mA, with a total exposure time of 8 s. The Dose Area Product (DAP) was recorded at 901 mGy*cm² to ensure radiological safety protocols. Patient positioning was strictly controlled using laser guides, with the mid-sagittal plane perpendicular to the floor and the Frankfort plane parallel to the floor, while the patient remained in a stable standing position (bipedestation).

#### CBCT image analysis and standardization

**Fig. 2:**
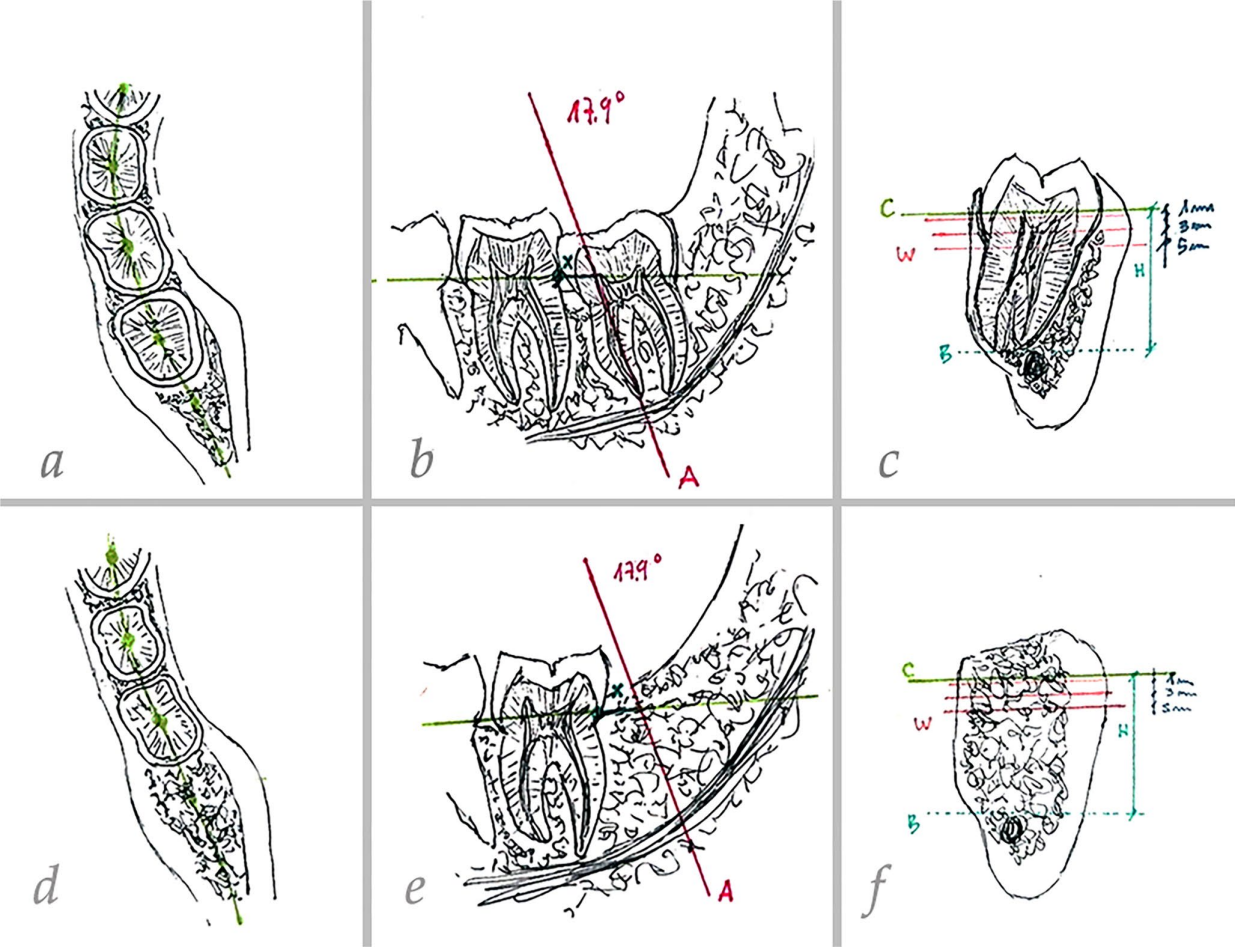
Illustrative scheme of dimensional bone changes measurements. (**a**–**c**) Baseline CBCT (T0): (**a**) coronal reference plane (plane C) passing through the center of the teeth; (**b**) sagittal cross-section obtained by lowering plane C to the level of the CEJ of the mandibular second molar. Line A was then drawn at a defined inclination along the long axis of the mandibular third molar to be extracted, intersecting the apex of the mesial root (point B). A fixed horizontal distance from the CEJ of the mandibular second molar to line A was also recorded to allow reproducibility in the postoperative scan; (**c**) horizontal sections generated at 1, 3, and 5 mm apical to plane C for ridge width measurements; vertical bone height measured from plane C to point B. (**d**–**f**) post-extraction CBCT at 12 weeks (T1), with plane C and line A reproduced at the same inclination and at the same CEJ-to-line A distance as at baseline, ensuring that measurements were performed at identical anatomical locations

CBCT scans were analyzed using ImplaStation software (*version 5.2119.1130*). Cross-sectional images from T0 and T1 were compared using a reproducible coordinate system based on stable anatomical landmarks to ensure that measurements were performed at identical locations over time [29, 34]. The method for establishing the reference planes, standardized cross-sections, and measurement points (Lines A, B, C) for both vertical (H) and horizontal (W) dimensional changes is fully illustrated in Fig. 2.

### Primary outcomes

#### Vertical bone H (ΔH)

The primary objective and the basis for the statistical power of this trial was the calculation of the dimensional change in vertical bone height (ΔH) between T0​ and T1​. All measurements were performed by a single, blinded operator (L.J.-A.) to eliminate inter-examiner variability.

Vertical Bone Height (ΔH) was defined as the distance (in mm) measured along Line A, extending from the horizontal reference at the CEJ level (Plane C) to the most apical point of the socket (Point B). A negative value in ΔH indicates bone gain or preservation, while a positive value indicates bone loss.

### Secondary outcomes

#### Exploratory bone outcomes

- *Horizontal Bone W (ΔW)*: Measured (in mm) at the three standardized horizontal levels apical to Line C (1 mm, 3 mm, and 5 mm).
- *Bone Density (Mean Gray Value)*: To evaluate bone maturation and mineral density—addressing the quality of the neoformed tissue—a densitometric analysis was performed at the same three standardized levels (1, 3, and 5 mm apical to Line C). Following the methodology of Wang et al. [35], grayscale values were recorded using ImageJ/Fiji software (National Institutes of Health, Bethesda, MD, USA), a tool widely validated for bone density assessment in both orthopedic and dental research [36, 37]. A circular Region of Interest (ROI) with a 4 mm diameter (area: 12.57 mm²) was placed in the center of the socket al.ong its long axis to average the neoformed bone trabeculae and minimize radiographic noise [38]. It is important to acknowledge that CBCT grayscale values are device-specific and should not be interpreted as standardized bone density measurements (Hounsfield Units). Therefore, these values were utilized as relative indicators of bone mineralization within the standardized parameters of this study protocol. Additionally, baseline (T0​) density measurements were not included in the analysis, as the post-extraction site at that stage consisted of non-mineralized components (blood clot and collagen scaffold), providing no radiopaque reference for bone mineral density.

#### Clinical variables

Clinical Variables were measured at baseline, 1, 12, 24, 48, 72 h, and 7 days postoperatively:

1. *Pain and Analgesic Consumption*: Pain intensity was recorded using a Visual Analog Scale (VAS) (0 to 10). The total number of analgesic tablets consumed over the first seven days was also recorded.
2. *Swelling*: Evaluated using a VAS at the specified time points.
3. *Trismus*: Objectively assessed by measuring the maximum interincisal opening (mm) at the specified time points [39–41].

### Statistical Analysis

Statistical analysis was performed using IBM SPSS Statistics version 24.0 (IBM Corp., Armonk, NY, USA). Means and standard deviations were calculated for numerical variables in both experimental groups. Data normality was assessed using the Kolmogorov–Smirnov test.

For variables demonstrating a normal distribution (*p* > 0.05), intergroup comparisons of mean dimensional changes (primary outcome ΔH and exploratory ΔW) and bone density (Mean Gray Value) were performed using the unpaired Student’s t-test. Between-group mean differences together with their corresponding 95% confidence intervals were calculated for all outcomes.

Repeated measures outcomes over time, such as postoperative pain and swelling scores, were analyzed using repeated-measures ANOVA with appropriate post hoc multiple comparison tests to evaluate temporal changes. In addition, between-group comparisons at each postoperative time point were performed using the independent samples Student’s t-test, and mean differences with 95% confidence intervals were calculated to facilitate clinical interpretation.

For datasets that did not meet assumptions of normality (*p* < 0.05) or for ordinal variables, intergroup comparisons were conducted using the Mann–Whitney U test. Categorical variables, including postoperative infection or complication rates, were evaluated using the Chi-square test or Fisher’s exact test, as appropriate.

Additionally, CBCT grayscale values were recorded at standardized regions of interest (ROIs) located 1, 3, and 5 mm apical to the cemento-enamel junction along the long axis of the socket. Between-group comparisons were performed using independent samples Student’s t-test. Welch correction was applied when homogeneity of variance was not met. Mean differences (SIM – Control) and corresponding 95% confidence intervals were calculated. These densitometric and secondary dimensional/clinical analyses were considered exploratory.

All CBCT measurements were performed by a single trained and blinded examiner (L.J.-A.) using a strictly standardized and reproducible protocol based on fixed anatomical reference points and identical image orientation at baseline and follow-up. This methodological standardization was applied to minimize intra-observer variability. However, formal intra-observer reproducibility (e.g., repeated measurements and intraclass correlation coefficient analysis) was not assessed as part of the original study design.

## Results

### Patient flow and baseline characteristics

A total of 40 patients were assessed for eligibility and subsequently randomized in a 1:1 ratio to the Test Group (collagen sponge + 1.2% SIM gel; *n* = 20 sockets) or the Control Group (collagen sponge + placebo gel; *n* = 20 sockets).

**Fig. 3:**
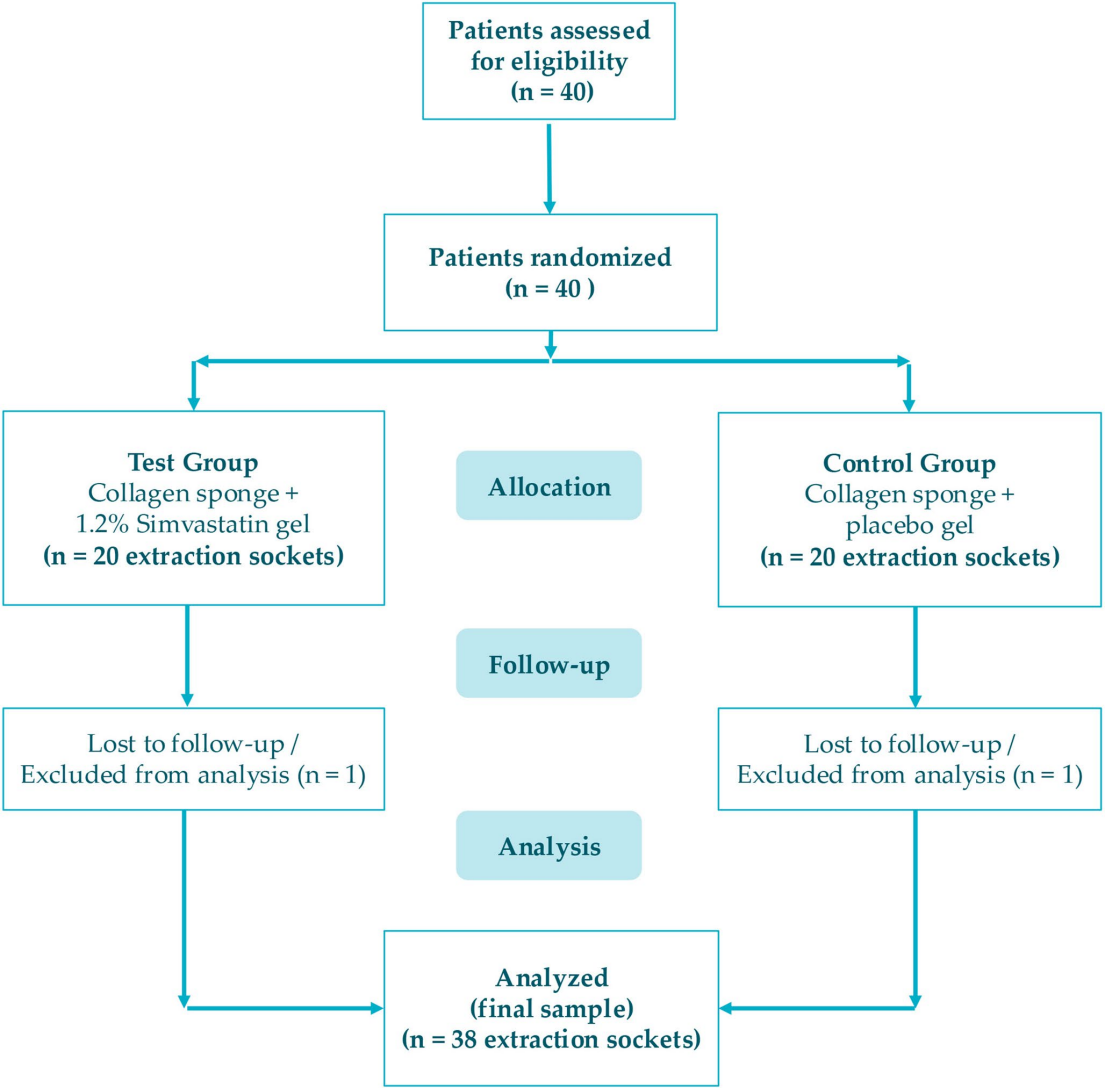
CONSORT flow diagram of the randomized clinical trial representing the flow from assessment for eligibility through randomization, allocation, follow-up, and analysis, detailing the two patients lost to follow-up

The trial execution is detailed in the CONSORT flow diagram (Fig. 3). All 40 patients completed the short-term clinical follow-up (0 h to 7 days) and were included in the secondary and exploratory outcome analyses (pain, swelling and trismus). However, two patients (one from the test group and one from the control group) were lost to follow-up at the 12-week visit because they did not attend the final CBCT examination. Therefore, clinical variables were analyzed for the total sample (*n* = 20 per group), whereas the final sample used for the radiographic analysis (primary and exploratory bone outcomes at 12 weeks) comprised 38 extraction sockets (*n* = 19 per group) with complete data available, following a complete-case analysis approach.

The demographic and surgical characteristics of the overall sample (*n* = 40 at randomization) are presented in Table 1. The sample was predominantly female, and the majority of extractions (55.00%) were performed on the mandibular right third molar. Regarding surgical variables, the Bayonet incision was the most frequently used (82.50% of cases), and the most common type of ostectomy required was the combination of mesial, vestibular, and distal bone removal (47.50%). Other descriptive variables, including tooth sectioning and surgery time, are fully detailed in Table 1.

**Table 1:** Descriptive analysis of patient- and surgery-related variables

Descriptive Variable	Subtipe	*n*	%
Sex	Male	14	35.00
	Female	26	65.00
Location	Mandibular left third molar	18	45.00
	Mandibular right third molar	22	55.00
Type of incision	Linear	7	17.50
	Bayonet	33	82.50
Periosteal tear	Yes	13	32.50
	No	27	67.50
Ostectomy	No ostectomy	2	5.00
	Mesial and vestibular	8	20.00
	Mesial, vestibular, and distal	19	47.50
	Mesial, vestibular, distal, and occlusal	11	27.50
Tooth sectioning	Yes No	16 24	40.00 60.00
Surgery time	< 30 min	24	60.00
	30–45 min	16	40.00

### Primary outcomes: vertical bone height (ΔH)

Dimensional changes in mandibular bone height and width were assessed 12 weeks after surgical extraction of mandibular third molars, following topical application of 1.2% SIM versus no application. Dimensional changes in bone height (ΔH) were analyzed individually, revealing a mean gain in bone height (− 0.30 ± 1.79 mm) in sockets treated with 1.2% topical SIM gel (Test Group, *n* = 19), compared to a mean reduction in height (1.28 ± 1.50 mm loss) observed in the Control Group (*n* = 19). The SIM group showed a mean vertical change of − 0.30 ± 1.79 mm compared with 1.28 ± 1.50 mm in the control group. The between-group mean difference (SIM − control) was − 1.58 mm (95% CI: −3.26 to − 0.73 mm; *p* = 0.006).

### Secondary outcomes

#### Exploratory bone outcomes

Horizontal Bone Width (ΔW): Regarding exploratory changes in bone width (ΔW), dimensional changes were measured at 1, 3, and 5 mm apical to reference line C. The mean loss in width at 1 mm (ΔW1​) was 1.17 ± 2.36 mm for the Test Group vs. 0.81 ± 1.69 mm for the Control Group (*p* = 0.594). Neither the dimensional changes at 1 mm nor at 3 mm (ΔW3​) or at 5 mm (ΔW5​) achieved statistical significance between the groups. For horizontal ridge width changes, the between-group mean differences were small and not statistically significant, with all 95% confidence intervals crossing zero (Table 2).

**Table 2:** Dimensional changes in alveolar ridge height and width at 12 weeks post-extraction

	*n*	ΔHeight (ΔH) m ± SD	ΔWidth 1 mm (ΔW1) m ± SD	ΔWidth 3 mm (ΔW3) m ± SD	ΔWidth 5 mm (ΔW5) m ± SD
Test	19	-0.30 ± 1.79	1.17 ± 2.36	1.18 ± 0.56	0.56 ± 1.31
Control	19	1.28 ± 1.50	0.81 ± 1.69	0.46 ± 1.16	0.11 ± 2.01
*p*-value*		**0.006**	0.594	0.354	0.463
Mean difference (95% CI)		**-1.58** (− 3.26 to − 0.73)	0.21 (− 0.68 to 1.11)	-0.22 (− 0.90 to 0.46)	0.38 (− 0.96 to 1.73)

ΔH: Change in alveolar bone Height; ΔW1: change in alveolar bone width at 1, 3, 5 mm from the reference, m: mean; SD: standard deviation. * Student’s t-test. For height changes (ΔH), negative values indicate bone gain or preservation, whereas positive values indicate bone loss. For width changes (ΔW), values represent the change relative to baseline measurements. Between-group mean differences are calculated as Test minus Control with 95% confidence intervals.

#### Bone density (mean gray value)

To further evaluate the quality of the newly formed bone, an exploratory densitometric analysis was performed by measuring absolute CBCT grayscale values at 12 weeks (T1​). Baseline (T0​) density measurements were not included in the comparative analysis as the socket was occupied only by non-mineralized components (blood clot and collagen scaffold) at that stage.

At the 12-week follow-up, grayscale values at 1 mm from the reference point were significantly higher in the SIM group compared with the control group (132.10 ± 87.75 vs. −14.72 ± 163.36; *p* = 0.0014), with a mean difference of 146.82 (95% CI: 60.54 to 233.10). At 3 mm, a numerical trend toward higher values was observed in the SIM group (113.93 ± 85.81 vs. 46.47 ± 139.40), although this did not reach statistical significance (*p* = 0.0825). No differences were detected at the 5 mm level (*p* = 0.9285). These findings suggest a localized coronal radiographic densitometric effect associated with topical simvastatin application at the end of the experimental period (Table 3).

**Table 3:** CBCT bone density (mean gray value) values at 12 weeks

Level	Group	Mean ± SD	Mean difference (SIM–Control)	95% CI	*p*-value
ROI 1 mm	Control (*n* = 19) SIM (*n* = 19)	–14.72 ± 163.36 132.10 ± 87.75	146.82	[60.54–233.10]	0.0014
ROI 3 mm	Control (*n* = 19) SIM (*n* = 19)	46.47 ± 139.40 113.93 ± 85.81	67.46	[-9.24–144.17]	0.0825
ROI 5 mm	Control (*n* = 19) SIM (*n* = 19)	112.23 ± 122.13 115.87 ± 126.36	3.64	[-78.12–85.41]	0.9285

Values are expressed as mean ± standard deviation.

Between-group comparisons were performed using independent samples t-test. Grayscale values represent absolute measurements at the 12-week follow-up (T1). Baseline (T0) values were not recorded for density analysis as the alveolar sockets contained only the collagen scaffold and blood clot, with no mineralized tissue present at that stage.

#### Clinical variables: pain, swelling and trismus

No statistically significant differences in postoperative pain or swelling were observed between the Test (SIM) and Control (Placebo) groups at any of the assessed time points, which included immediate assessment (0 h) and specific intervals up to 7 days postoperatively (see Tables 4 and 5). The analysis included all 40 randomized patients. The peak mean pain scores occurred at 24 h post-extraction for both the test group (4.38 ± 1.85) and the control group (4.58 ± 2.30), with no significant intergroup difference (*p* = 0.791). Similarly, the swelling scores peaked at 24 h (Test: 5.46 ± 1.19; Control: 5.71 ± 1.89; *p* = 0.674). Between-group comparisons of postoperative pain scores at each time point were performed using the independent samples Student’s t-test. The between-group mean differences were small at all evaluated intervals, and the corresponding 95% confidence intervals crossed zero, indicating no statistically or clinically relevant differences between the SIM and control groups (Table 4).

**Table 4:** Postoperative pain scores at different time points in the test and control groups

		n	m ± SD	Test vs. Control Mean difference	95% CI	*p*-value
Pain 0 h	Test	20	1.38 *±* 1.89	0.75	[-0.25–1.75]	0.135
	Control	20	0.63 ± 1.13			
Pain 1 h	Test	20	1.31 ± 1.93	0.02	[-1.09–1.13]	0.978
	Control	20	1.29 ± 1.51			
Pain 12 h	Test	20	3.15 ± 2.34	-0.18	[-1.81–1.45]	0.843
	Control	20	3.33 ± 2.74			
Pain 24 h	Test	20	4.38 ± 1.85	-0.20	[-1.54–1.14]	0.791
	Control	20	4.58 ± 2.30			
Pain 48 h	Test	20	3.31 ± 1.54	-0.61	[-1.85–0.63]	0.393
	Control	20	3.92 ± 2.26			
Pain 72 h	Test	20	2.15 ± 2.07	-0.10	[-1.51–1.31]	0.902
	Control	20	2.25 ± 2.32			
Pain 7 d	Test	20	0.85 ± 1.51	0.18	[-0.83–1.19]	0.746
	Control	20	0.67 ± 1.63			

*m* mean, *SD* standard deviation, *CI* confidence interval. *Student’s t-test. Values are expressed as mean ± standard deviation. Between-group comparisons at each postoperative time point were performed using the independent samples Student’s t-test. Mean differences are calculated as Test minus Control with corresponding 95% confidence intervals

Similarly, postoperative swelling scores showed no significant between-group differences at any time point. Mean differences and 95% confidence intervals consistently crossed zero, suggesting comparable postoperative inflammatory responses between groups (Table 5).

**Table 5:** Postoperative swelling scores at different time points in the test and control groups

		*n*	m ± SD	Test vs. Control Mean difference	95% CI	*p*-value
Swelling 0 h	Test	20	1.00 ± 1.73	0.33	[-0.75–1.41]	0.483
	Control	20	0.67 ± 1.63			
Swelling 1 h	Test	20	1.15 ± 1.81	0.48	[-0.62–1.58]	0.592
	Control	20	0.67 ± 1.63			
Swelling 12 h	Test	20	3.15 ± 2.94	-0.20	[-1.93–1.53]	0.833
	Control	20	3.35 ± 2.44			
Swelling 24 h	Test	20	5.46 ± 1.19	-0.25	[-1.26–0.76]	0.674
	Control	20	5.71 ± 1.89			
Swelling 48 h	Test	20	4.46 ± 1.61	0.08	[-1.13–1.29]	0.900
	Control	20	4.38 ± 2.14			
Swelling 72 h	Test	20	2.77 ± 2.08	0.27	[1.24–1.78]	0.751
	Control	20	2.50 ± 2.6			
Swelling 7d	Test	20	0.69 ± 1.10	0.23	[-0.41–0.87]	0.487
	Control	20	0.46 ± 0.88			

*m* mean, *SD* standard deviation, *CI* confidence interval. *Student’s t-test.

Values are expressed as mean ± standard deviation. Between-group comparisons at each postoperative time point were performed using the independent samples Student’s t-test. Mean differences are calculated as Test minus Control with corresponding 95% confidence intervals.

Regarding trismus (Table 6), no significant differences were found between the SIM and placebo groups at baseline (44.58 ± 5.09 mm vs. (41.33 ± 5.48 mm; *p* = 0.442). At 7 days post-extraction, both groups showed a similar reduction in opening capacity, with no statistically significant differences observed (37.37 ± 9.38 mm for the test group vs. 36.61 ± 5.37 mm for the control group; *p* = 0.781), indicating a comparable pattern of functional recovery. Furthermore, no significant adverse reactions or complications were recorded across the study population. Only one case of wound dehiscence was reported as a postoperative complication.

**Table 6:** Maximum interincisal opening (trismus), measured in millimeters at baseline and 7 days post-extraction

Time Point	Group	*n*	Mean ± SD	95% CI	*p*-value
Baseline	Test Control	20 20	44,58 ± 5.09 41.33 ± 5.48	[42.14–47.02] [38.71–43.95]	0.442
7 days	Test Control	20 20	37.37 ± 9.38 36.61 ± 5.37	[32.87–41.87] [34.04–39.18]	0.781

*m* mean, *SD* standard deviation, *CI* confidence interval. *Student’s t-test.

Values are expressed as mean ± standard deviation. Between-group comparisons at each postoperative time point were performed using the independent samples Student’s t-test. Mean differences are calculated as Test minus Control with corresponding 95% confidence intervals.

## Discussion

The resorption of alveolar bone following tooth extraction is a well-documented phenomenon, with significant clinical implications for future prosthetic rehabilitation [2, 3]. This randomized clinical trial aimed to evaluate the efficacy of topical application of simvastatin (SIM) gel in mitigating this resorption after mandibular third molar extraction, a challenging anatomical site. The most significant finding of the present study is the demonstration of a statistically significant gain in alveolar bone height (primary outcome, ΔH = − 0.30 ± 1.79 mm) in sockets treated with 1.2% SIM gel compared to the placebo group (ΔH = 1.28 ± 1.50 mm loss; *p* = 0.006). This result supports the strong osteoinductive potential of topical SIM, further confirming its potential as a safe, cost-effective pharmacological alternative for ridge preservation.

The dimensional reduction in both height and width of the alveolar ridge may compromise the placement and stability of dental implants [4–6]. While various biomaterials and regenerative techniques have been developed to mitigate these effects [4, 6], they often involve higher costs, surgical complexity, and risk of postoperative complications [7]. Pharmacological approaches in this context that promote bone formation in a non-invasive and cost-effective manner have gained increasing interest [7, 14]. The present finding—a statistically significant gain in alveolar bone height—is consistent with previous clinical trials using the third molar extraction model. Degala and Bathija [28], in a split-mouth randomized trial, reported enhanced bone regeneration after the local application of simvastatin in bilaterally impacted third molars. Similarly, Tidke et al. [24] observed superior outcomes in bone healing with SIM gel versus powder, highlighting the efficacy of the gel formulation. Given that the third molar region represents an anatomically challenging site for regeneration due to structural and mechanical factors [9, 36], the statistically significant vertical gain found in this study reinforces the potential of topical SIM even in challenging clinical sites. Importantly, the present trial used a 12-week healing period, which is more appropriate for mandibular bone remodeling than the 8-week interval used in other trials [27], allowing a more reliable evaluation of osseous healing. In addition, the use of CBCT with a fixed anatomical reference (the distal aspect of the second molar) provided greater reproducibility and precision in dimensional assessment, in contrast to periapical radiographs used in other studies. This methodology, along with the strict inclusion criteria and standardized surgical protocol, strengthens the internal validity of the present findings.

Regarding bone width (ΔW), no statistically significant difference was found between the groups (*p* > 0.05). While the SIM group showed numerically less loss at the 3 mm and 5 mm levels (Table 2), the overall preservation was not significantly different. This result aligns with previous studies suggesting SIM enhances bone density and trabecular organization [12, 16, 17]. Notably, Cruz et al. reported significant multidimensional improvements following topical simvastatin application in extraction sockets of premolars. The difference in horizontal outcomes between our study and Cruz et al. [23] may be attributed to their use of a rigid physical barrier (polypropylene membrane) and a more favorable anatomical site. Unlike the premolar model, the retromolar area is characterized by high cortical density and constant mechanical tension from the buccinator and mylohyoid muscle insertions, factors that complicate regenerative stability [40, 41]. Consistent with previous reports indicating that ridge preservation outcomes are highly site-dependent (Hämmerle et al. [42] and Vignoletti et al. [43]), the findings of the present study should therefore be interpreted as specific to this anatomical model.

It is important to emphasize that while the exploratory densitometric findings are encouraging, the most clinically relevant result of this trial is the significant preservation of vertical bone height. Although the mean vertical bone change observed in the test group was limited (-0.30 mm), the intergroup difference in ΔH (1.58 mm) may be clinically relevant in implant dentistry. Vertical dimensional preservation of this magnitude can reduce the need for additional augmentation procedures, facilitate the use of standard-length implants, and improve prosthetically driven implant positioning, particularly in sites with limited residual bone height. Nevertheless, the use of third molar extraction sites, while offering a controlled and reproducible model, inherently limits the extrapolation of these findings to esthetic zones or load-bearing posterior regions. Accordingly, the results should be interpreted within the context of this experimental model [11, 28].

Complementing this primary outcome, our exploratory Bone Density (Mean Gray Value) analysis revealed a significant increase in absolute grayscale values at the 1 mm coronal level in the SIM group (*p* = 0.0014) at the end of the 12-week period. To ensure the validity of these findings, we utilized ImageJ/Fiji software, a methodology widely recognized for bone density assessment [35–37]. This radiographic surrogate of bone quality is supported by the high correlation between CBCT grayscale values and bone microarchitecture (Micro-CT) reported by Choi et al. [38], suggesting that SIM not only limits vertical resorption—the primary objective of our study—but may also enhance the mineralization quality of the neoformed tissue even in a “high-difficulty” scenario.

Regarding the inflammatory response, no statistically significant differences were found in postoperative pain or swelling. These results are consistent with Diniz et al. [29], suggesting that while SIM may modulate bone microarchitecture, its clinical impact on patient-reported outcomes may be masked by surgical variability. The safety of the topical application was confirmed, with no serious adverse events, providing further clinical validation for local SIM delivery [12, 19].

Among the limitations of this study is the relatively small sample size. Although adequately powered for the primary outcome (ΔH), the study was underpowered for exploratory parameters (width, density, and clinical variables). In addition, all CBCT measurements were performed by a single trained and blinded examiner using a strictly standardized protocol with stable anatomical reference points; however, formal intra-observer reproducibility (e.g., repeated measurements and intraclass correlation coefficient analysis) was not assessed as part of the original study design. This represents a limitation that should be addressed in future research through predefined calibration and reliability analyses to further strengthen methodological robustness.

The 12-week follow-up provides a robust proof-of-concept for the pharmacological efficacy of SIM during the critical early healing phase [43]. Future research should investigate the potential synergistic effects of 1.2% SIM gel combined with bone grafts (xenografts) and resorbable membranes to optimize multidimensional preservation, building upon the encouraging, though initial, vertical and qualitative findings presented here.

## Conclusions

The results of this randomized clinical trial demonstrate that the topical application of 1.2% simvastatin gel, combined with a collagen carrier, significantly improved vertical alveolar bone height preservation at 12 weeks, achieving the primary endpoint of the study. Exploratory findings regarding bone width and densitometric mineralization patterns suggested differences in radiographic mineralization patterns; however, these results should be interpreted cautiously. At present, this approach should be considered a promising and accessible adjunctive strategy rather than a substitute for established regenerative procedures. Further studies with larger samples and in combination with conventional biomaterials are warranted to confirm its clinical applicability, particularly in esthetic and load-bearing regions.

## Data Availability

The data presented in this study are available on request from the corresponding author.
